# PLCL1 suppresses tumour progression by regulating AMPK/mTOR-mediated autophagy in renal cell carcinoma

**DOI:** 10.18632/aging.205085

**Published:** 2023-10-06

**Authors:** Zhou Pan, Jing Huang, Huajie Song, Yusha Xiao, Ting Liu, Yan Zeng, Hengcheng Zhu, Kang Yang

**Affiliations:** 1 Department of Urology, Renmin Hospital of Wuhan University, Wuhan 443002, P.R. China; 2 Division of Nephrology, Renmin Hospital of Wuhan University, Wuhan 443002, P.R. China; 3 Department of Cardiovascular Surgery, Zhongnan Hospital of Wuhan University, Wuhan 443002, P.R. China

**Keywords:** renal cell carcinoma, autophagy, PLCL1, AMPK/mTOR pathway

## Abstract

Autophagy has been increasingly recognized as a critical regulatory mechanism in the maintenance of cellular homeostasis. A previous study showed that phospholipase C-like protein 1 (PLCL1) is associated with lipid metabolism in renal cell carcinoma (RCC). However, it is unclear whether PLCL1 regulates autophagy, thereby influencing the progression of RCC. Bioinformatics analysis of five microarray datasets revealed that expression of PLCL1 is decreased in tumours and is positively correlated with prognosis in RCC patients. Three independent public datasets, clinical RCC tissues and RCC cell lines, were validated using real-time qPCR, western blotting and immunohistochemistry. Using wound healing and transwell assays, we observed that elevated PLCL1 levels decreased the migratory distance and the invasive number of 786-O and ACHN cells, but PLCL1 knockdown reversed these changes in 769P cell lines compared to those in controls. The results of flow cytometry analysis indicated that PLCL1 promotes apoptosis. Moreover, transcriptional analysis based on stable overexpression of PLCL1 in 786-O cells revealed that PLCL1 is related to autophagy, and western blotting and autophagic experimental results further verified these findings. Mechanistic investigations confirmed that PLCL1 activates the AMPK/mTOR pathway and interacts with decidual protein induced by progesterone (DEPP). Collectively, our data suggest that PLCL1 functions as a suppressor of RCC progression by activating the AMPK/mTOR pathway, interacting with DEPP, initiating autophagy and inducing apoptosis. PLCL1 may be a promising therapeutic target for the diagnosis and treatment of ccRCC patients.

## INTRODUCTION

Renal cell carcinoma (RCC) is the third most frequent genitourinary malignancy, accounting for at least 400,000 new cases and 175,000 deaths each year worldwide [1, 2]. The incidence rate of RCC is increasing, which may be due to early abdominal imaging detection in patients with gastrointestinal complaints [3, 4]. However, up to 17% of RCC cases become advanced, and distant metastases due to developing drug resistance to current therapy often occurs [1]. Therefore, a better understanding of the potential molecular pathogenesis of RCC is urgently needed to explore and seek satisfactory treatment for RCC patients.

Phospholipase C-like protein 1 (PLCL1), a member of the phospholipase C family, is expressed in a range of organs, including the brain, heart, and kidney, and unlike phosphatidylinositol 4,5-bisphosphate (PIP2) and phosphatidylinositol (PI), exhibits catalytic activity [5]. A previous study indicated that PLCL1 mutation could deactivate the 1,4,5-trisphosphate (IP3)-mediated calcium pathway, a crucial signal in regulating mechanical sensing of bone cells, altering the hip bone size in females [6]. Moreover, a cohort investigation revealed that an underlying genetic susceptibility due to PLCL1 was correlated with idiopathic inflammatory myopathies in different ethnic populations of Chinese Han [7]. Recently, it has been demonstrated that PLCL1 induces abnormal lipid metabolism in tumour cells by interacting with metabolism-related gene uncoupling protein 1 (UCP1), repressing RCC progression [8]. These studies all support the contention that PLCL1 is as an important regulator in disease.

Macroautophagy/autophagy is a primary intracellular catabolic process and a key cellular regulatory mechanism that degrades damaged components in cells and recycles or removes dysfunctional organelles [9]. It is characterized by the formation of cellular double membrane vesicles called autophagosomes, which sequester cytoplasmic organelles before fusing with lysosomes for degradation [10]. Autophagy has attracted much attention due to its important role in regulating multifarious pathophysiological processes, especially in urinary system diseases, including renal ischaemia reperfusion injury (IRI), renal fibrosis, and urological tumours. Fibroblast growth factor 10 and lipocalin-2 protect mice against renal IRI via autophagy activation mediated by the inflammatory response [11, 12]. Autophagy-related gene (ATG)-5-mediated autophagy deficiency in proximal tubules exacerbates the progression of renal fibrosis [13]. Moreover, AMP-activated protein kinase (AMPK) activated by intra- and extracellular stimulation decreases the expression of mammalian target of rapamycin (mTOR), induces the expression of ATGs and the conversion of MAP1LC3B (LC3)-I to LC3B-II and promotes RCC, bladder and prostate cell apoptosis [14–16]. To date, more than 50 genes associated with autophagy have been associated with a series of diseases; however, whether and how PLCL1 affects RCC progression by regulating autophagy remain unclear.

In this study, PLCL1 was identified using mRNA microarrays in the Gene Expression Omnibus (GEO) derived from RCC and adjacent normal tissues. Four independent datasets and The Cancer Genome Atlas (TCGA) databases as well as RCC patient tissues were then examined to validate aberrant expression of PLCL1. Moreover, the capacity of PLCL1 to suppress tumour progression was explored and confirmed through cell proliferation and invasion, apoptosis, xenograft and orthotopic transplantation models. Notably, transcriptome sequencing revealed the potential mechanism of PLCL1 as activation of AMPK/mTOR-mediated autophagy through an interaction with decidual protein induced by progesterone (DEPP). These findings offer a novel tumour suppressor mechanism for PLCL1, suggesting that PLCL1 represents a therapeutic biomarker for RCC treatment.

## MATERIALS AND METHODS

### Data collection, pre-processing, and bioinformatics analysis

Microarray datasets GSE36895, GSE53757, GSE68417, GSE66270, GSE16441, GSE781, and GSE634 were downloaded from the GEO database (http://www.ncbi.nlm.nih.gov/geo/). Each dataset was normalized and analysed using the Limma package, and transcriptional data from TCGA (https://portal.gdc.cancer.gov/) were used with the EdgeR package. Only differentially expressed genes (DEGs) with |log_2_ fold change| > 1 and *P* value < 0.05 in each microarray between RCC tissues and normal samples were included in this study for further analysis.

### RCC specimens and patients

Thirty-five pairs of RCC specimens between tumour and adjacent normal samples were collected from patients diagnosed with RCC at Renmin Hospital of Wuhan University (Hubei, China). Fresh tissues obtained during surgery were stored in liquid nitrogen for subsequent RNA extraction and western blots. This study was approved by the Research Ethics Committee of Renmin Hospital of Wuhan University. Informed consent was obtained from all patients (No. 20190420).

### Cell lines and reagents

The normal human renal cell line HK2 and human renal cancer cell lines ACHN, 786-O, 769P and Caki-1 were purchased from the China Centre for Type Culture Collection (Wuhan, China) and The American Type Culture Collection (USA). All cell lines were cultured based according to the manufacturers’ instructions. The expression lentivirus of PLCL1, small interfering RNA (siRNA) of PLCL1 (siRNA1 and siRNA2 target sequences: CCGGCCAAATTCTCGCATT, GCGCAAATACAAAGGGCAT), expression lentivirus of DEPP and corresponding controls were purchased from GeneChem (Shanghai, China) and Genecreate (Wuhan, China), respectively. The autophagy inhibitor 3-methyladenine (3-MA) and the autophagy inducer rapamycin were purchased from MedChemExpress (Shanghai, China).

### Cell infection and transfection

ACHN, 786-O and 769P cell lines were cultured in complete medium with 10% foetal bovine serum (FBS, ScienCell, USA) and 1% penicillin–streptomycin (Gibco, USA) and maintained in humidified conditions of 5% CO_2_ at 37°C overnight. Then, ACHN, 786-O, and 769P cells were transfected with lentivirus, siRNA and corresponding controls using Lipofectamine 3000 (Invitrogen, USA) in complete medium with polybrene (Sigma, USA) for 24 h according to the manufacturers’ protocols. Then, the cells were harvested to extract total RNA and protein to verify the transfection efficiency using real-time (RT)–qPCR and western blotting after 48-72 hours. After transfected for 72 hours, cells were subjected to subsequent experiments on cell proliferation, migration, apoptosis and autophagy. All subsequent cellular experiments were performed in triplicate.

### Real-time quantitative PCR

Total RNA was extracted from ccRCC samples and cells using TRIzol reagent (Invitrogen, USA), and the purity and concentration were assessed using a NanoDrop ultramicro ultraviolet spectrophotometer (NanoDrop, USA). Then, these analytes were reverse transcribed into cDNA using the PrimeScript RT Reagent Kit with gDNA Eraser (Takara, Kusatsu, Japan). RT–qPCR was conducted using SYBR Green mix (Thermo Fisher Scientific, USA). The primer sequences were as follows:

**Table:** 

PLCL1:	Forward, 5′-AAAGTCCGGCCAAATTCTCG-3′;
	Reverse, 5′-TTTCCGTGTTTTTCCCCAGTC-3′;
DEPP:	Forward, 5′-GTGAGGTCTATATCTCGACTGGC-3′;
	Reverse, 5′- ACTGAAACGTGCGGTGATGT-3′;
GAPDH:	Forward, 5′-AATCCCATCACCATCTTCCAG-3′;
	Reverse, 5′-GAGCCCCAGCCTTCTCCAT-3′.

### Western blots

Western blotting analysis was performed as previously described [17]. Briefly, cellular protein was lysed in lysis buffer (Servicebio, Wuhan, China), and the concentration of protein was determined using a BCA Protein Assay Kit (Thermo Fisher Scientific, USA). Then, 50 μg of each sample was separated using 10% SDS–PAGE and transferred to polyvinylidene fluoride (PVDF) membranes. After blocking in 5% milk, the membranes were incubated with primary antibodies (Supplementary Table 1) overnight. Finally, the membranes were incubated with secondary antibody (LI-COR Biosciences, USA) and imaged using a two-colour infrared imaging system (Odyssey, USA).

### Immunohistochemistry and immunofluorescence staining

Four-millimeter-thick sections of paraffin-embedded ccRCC patient and mouse tissues were stained with haematoxylin and eosin. Immunohistochemistry (IHC) staining was conducted according to the manufacturers’ protocols. Briefly, after deparaffinization, each slide was incubated with primary antibodies (Supplementary Table 1) at 4°C overnight followed by incubation with HRP-conjugated secondary antibody for 30 minutes. Finally, each specimen was stained with 3,3-diaminobenzidine tetrahydrochloride (Maixin, Fuzhou, China). For immunofluorescence staining, each slide was incubated with Alexa Fluor secondary antibody (Cell Signaling Technology, USA), and the nuclei of each slide were stained with DAPI for 5 min. Images of each slide were acquired using a microscope (Olympus, Tokyo, Japan).

### Cell proliferation assay

A Cell Counting Kit-8 (CCK8, Japan) and Edu kit (Beyotime, Shanghai, China) was used to measure the proliferation rate of ACHN, 786-O and 769P cells according to the manufacturer’s protocols.

### Wound healing and transwell assays

ACHN, 786-O and 769P cells were seeded into 6-well plates. When the cells reached 80% confluence, a 200 μl yellow pipette tip was used to create a wound, and the cells were subsequently incubated in serum-free medium. Images were acquired 0 and 24 h after wounding. For the transwell and invasion assays, a density of 6×10^4^ cells was seeded into the upper chamber of each well in Matrigel (Becton, Dickinson and Company, USA) and incubated in serum-free medium. After 24 h, the cells that had invaded the membrane were stained with 0.05% crystal violet.

### Flow cytometry analysis of apoptosis

ACHN, 786-O and 769P cells were infected with lentivirus and siRNA. After 24 h, the cells were collected and stained with Annexin V-FITC and propidium iodide (PI) and analysed using flow cytometry according to the manufacturer’s instructions (BD Biosciences, USA).

### High-throughput RNA sequencing

First, 2 μg of total RNA from 786-O cells with stable overexpression and vector were utilized for r-stranded RNA sequencing library preparation using the KCTM Stranded mRNA Library Prep Kit for Illumina (NO. DR08402, Seqhealth, Wuhan, China) following the manufacturer’s instructions as previously described [18, 19]. Briefly, after assessing the material using a Nanodrop spectrophotometer (Thermo Fisher Scientific, USA) and confirmation by 1.5% agarose gel electrophoresis, 200- to 500-bp PCR products were cleansed, quantified, and sequenced using a HiSeq X10 sequencer (Illumina). Then, the raw sequencing data of each sample were screened using Trimmomatic (version: 0.36) and mapped to the human genome (GRCh38.p13) using Tophat (version 2.1.0). The gene expression in each sample was determined using Cufflinks (version 2.21). Pathway enrichment results with a *P* value < 0.05 were obtained using the Database for Annotation, Visualization and Integrated Discovery (DAVID) version 6.8 (https://david.ncifcrf.gov).

### Transmission electron microscopy

Cells were fixed in 2.5% glutaraldehyde in 0.1 M sodium cacodylate buffer (pH 7.2) and then postfixed in 1% phosphate-buffered osmium tetroxide at pH 7.4. Subsequently, all specimens were dehydrated, embedded in epoxy resin, sectioned and double-stained with lead citrate and uranyl acetate. Finally, each section was imaged using transmission electron microscopy (Hitachi, Tokyo, Japan).

### Adenoviral transfection

Adenovirus with human GFP-mRFP-LC3 was purchased from HanBio (Shanghai, China) to detect autophagosomes and autolysosomes in ACHN, 786-O and 769P cells according to the manufacturer’s protocols. Briefly, cells were seeded into confocal culture dishes and transfected with the adenovirus for 24 h. After washing with phosphate-buffered saline, autophagic flux was imaged with a confocal microscope (Olympus, Tokyo, Japan).

### Immunoblot and coimmunoprecipitation

The coimmunoprecipitation (CoIP) reagent of the Pierce™ c-Myc-Tag Magnetic IP/Co-IP Kit (Thermo Fisher, USA) was utilized to examine the interaction based on the manufacturer’s protocols. Briefly, 5 μg of PLCL1 antibody (Abcam, USA), anti-myc antibody and IgG were covalently crosslinked with cell lysates at 4°C overnight. The immune complex was then incubated with Sepharose protein A/G magnetic beads for 1 hour, and the unbound immune complexes were removed. The bound immune complexes were eluted using a low-pH elution buffer and analysed by immunoblotting.

### Orthotopic and xenograft tumour model

All experimental procedures were approved by the Ethical Committee of Institutional Animal Care and Treatment Committee of Renmin Hospital of Wuhan University. Orthotopic and xenograft tumour models were constructed in male Balb/c nude mice (20-25 g) purchased from the Charles River Animal Technology Company (Beijing, China). Briefly, to establish an orthotopic renal tumour model, 786-O cells stably overexpressing PLCL1 and vectors were injected directly into the kidneys of mice after exposing the right kidney using a 1 ml syringe. After 28 days, all mice were scanned using positron emission computed tomography (TransPET Discoverist 180, Wuhan, China) and sacrificed for further study. To construct the xenograft tumour model, 786-O cells stably overexpressing PLCL1 and vectors were directly injected into the right armpit. Then, the mice were sacrificed, and the tumours were removed for further assessment 28 days later. Tumour volume in each group of mice (*n* = 5) was calculated as (length × width^2^)/2.

### Statistical analysis

GraphPad Prism 7 (GraphPad Software, USA) was applied to analyse differences between two groups (Student’s *t* test) and multiple groups (one-way ANOVA). The relationship between PLCL1 and DEPP was examined using Pearson correlation. Univariate and multivariate analyses were performed to determine the relationship between the expression of DEGs and patient survival time, and the Kaplan–Meier method was utilized to analyse the overall survival of patients in TCGA using R software (Version 3.6.1). Data in our study are shown as the mean ± error, and all experiments were completed three times. *P* values less than 0.05 were considered significant.

### Availability of data and materials

The datasets during and/or analysed during the current study are available from the corresponding author on reasonable request.

## RESULTS

### PLCL1 expression is downregulated and related to prognosis in RCC

**Figure 1:**
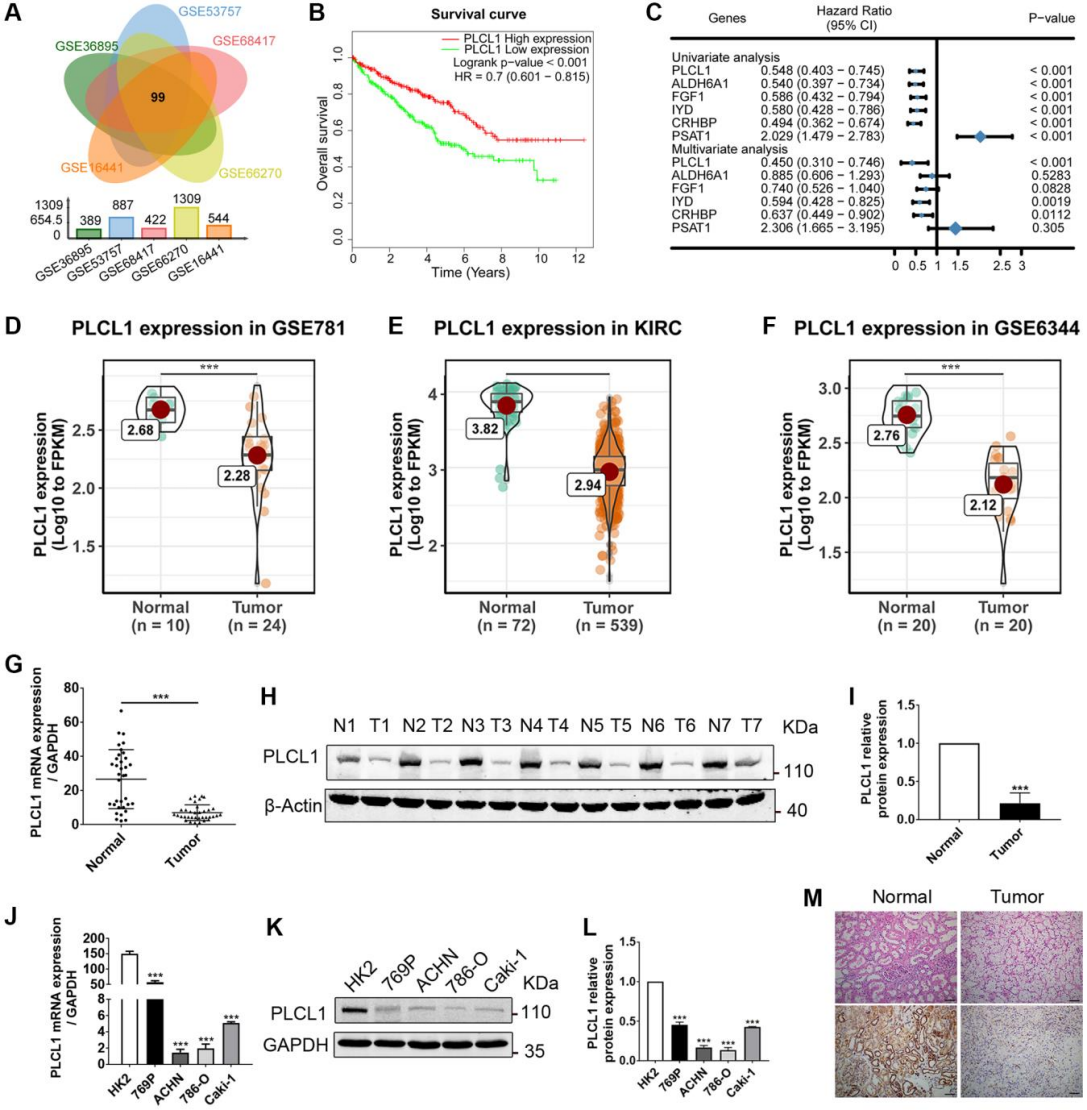
**PLCL1 expression is downregulated in RCC tumour tissues and closely related to RCC patient prognosis.** (**A**) Differentially expressed genes (DEGs) in GSE36895, GSE53757, GSE68417, GSE66270, and GSE16441 were intersected using Venn diagrams. (**B**) Kaplan–Meier curve of RCC patients grouped based on median levels of PLCL1. HR, hazard ratio. (**C**) Univariate and multivariate analyses showing the relationship between significantly intersected DEG levels and RCC patient survival. CI, confidence interval. (**D**–**F**) Representative expression levels of PLCL1 in tumour samples and paired adjacent normal tissues (PANT) from public datasets. (**G**, **H**) Representative RT–qPCR and western blot analysis of PLCL1 in RCC tissue and PANT. (**I**) Quantification of PLCL1 protein levels. β-actin was used as a loading control. (**J**) Representative RT–qPCR analysis of PLCL1 in normal human kidney HK2 cells and tumour cells. (**K**, **L**) Representative western blots and quantification of PLCL1. GAPDH was used as a loading control. (**M**) Representative HE and immunohistochemistry analysis of PLCL1 in RCC tissue and PANT. Scale bar: 100 μm. Data are shown as the mean ± SE from three independent experiments. Student’s *t* test was performed to determine statistical significance between two groups. Scale bar: 100 μm. ^*^P < 0.05; ^**^P < 0.01; ^***^P < 0.001.

To identify important DEGs in the pathogenesis of RCC, DEGs were analysed and intersected from five GEO datasets using R software. As shown in Figure 1A, the results of the Venn diagram showed that 99 important genes were significant in all five datasets. Interestingly, hierarchical clustering of the five datasets using the 99 DEGs separated RCC from normal samples (Supplementary Figure 1). The Kaplan–Meier curve showed that 6 genes were significantly related to prognosis in RCC patients (*P* value < 0.001, Figure 1B, Supplementary Figure 2). The results of univariate and multivariate regression revealed that three genes named PLCL1, CHRBP and IYD could serve as independent prognostic biomarkers for RCC (Figure 1C). In addition, our bioinformatics findings were validated in three independent GEO datasets, indicating that expression levels of PLCL1 are downregulated in RCC patients (Figure 1D–1F). To further verify the findings from the public datasets, RCC and adjacent normal specimens as well as tumour and normal kidney cells were used to evaluate mRNA and protein expression levels of PLCL1. As shown in Figure 1G–1I and Figure 1M, compared to the normal group, PLCL1 expression in tumour samples was significantly decreased at the mRNA and protein levels, and similar results were observed between HK2 and RCC cell lines (Figure 1J–1L). These data confirm an important role for PLCL1 in the pathogenesis of RCC.

### PLCL1 functions as a tumour suppressor of RCC migration and invasion *in vitro*

**Figure 2:**
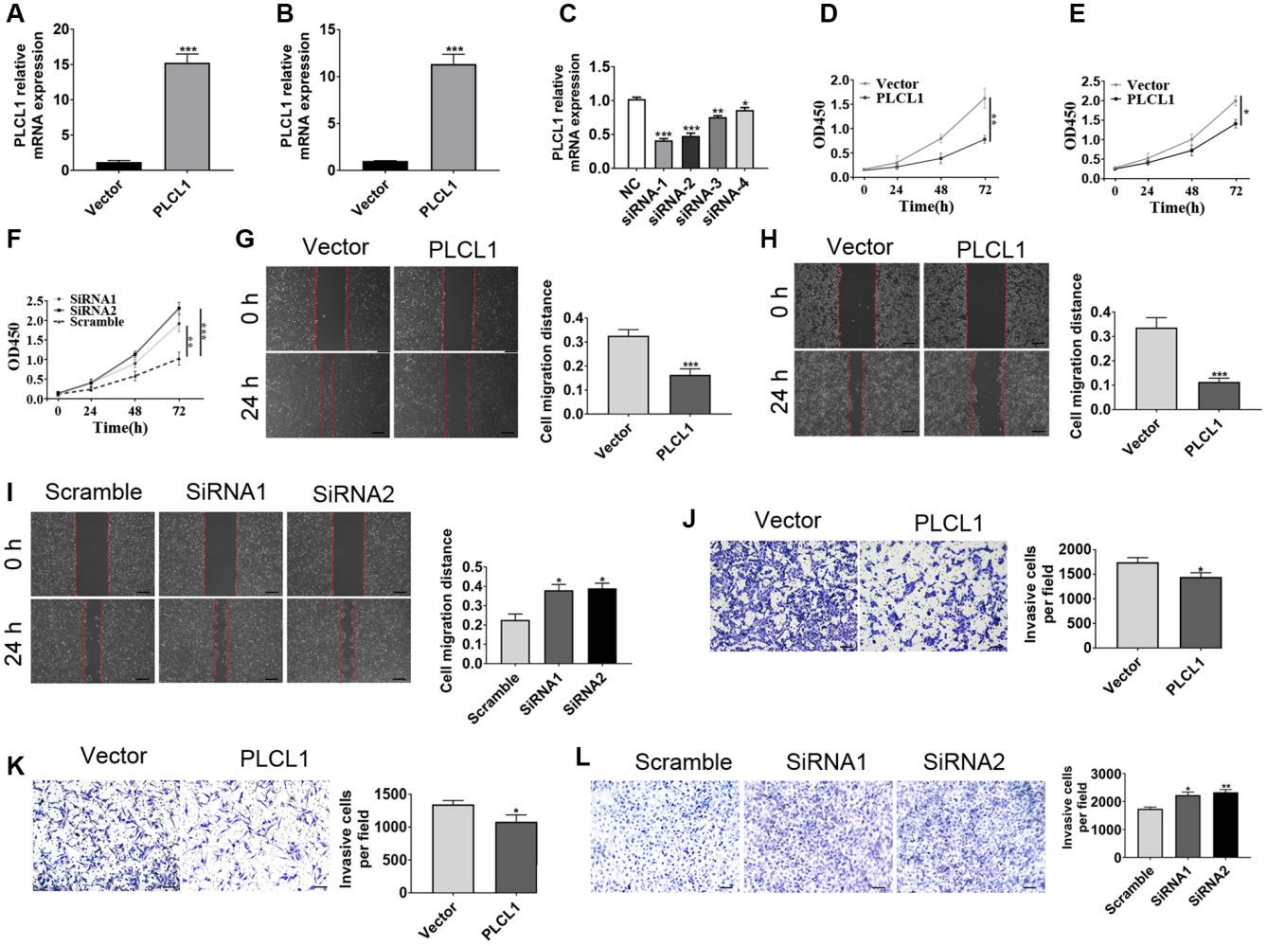
**PLCL1 inhibits RCC cancer cell migration and invasion.** (**A**, **B**) Representative RT–qPCR analysis of 786-O and ACHN cells transfected with lentiviral vector encoding PLCLA and lentivirus vector. (**C**) Representative RT–qPCR analysis of 769P cells transfected with PLCL1 siRNA or scrambled control. (**D**–**F**) CCK8 assays were utilized to determine the proliferation of 786-O, ACHN and 769P cell lines. (**G**–**I**) Wound healing assays were used to explore the role of PLCL1 in RCC cells, and quantification analyses of the results were performed at 0 and 24 hours. Scale bar: 100 μm. (**J**–**L**) 786-O, ACHN and 769P cell lines with different treatments were examined by transwell assay. Scale bar: 100 μm. Data are shown as the mean ± SE from three independent experiments. Student’s *t* test was performed to determine statistical significance between two groups. Scale bar: 100 μm. ^*^P < 0.05; ^**^P < 0.01; ^***^P < 0.001.

To assess the function of PLCL1 in the progression of RCC, the ACHN, 786-O and 769P cell lines were utilized for functional experiments. ACHN and 786-O cells were transfected with lentivirus overexpressing PLCL1, whereas 769P was transfected with siRNA against PLCL1. Both the PLCL1 transfection efficiency in overexpression and knockdown cell were verified by RT–qPCR (Figure 2A–2C). The results of cell proliferation assays using CCK8 showed that PLCL1 overexpression significantly repressed the proliferation of ACHN and 786-O cells, while knockdown of PLCL1 reversed this alteration in 769P cells (Figure 2D–2F). The results of wound healing assays indicated that elevated levels of PLCL1 decreased the migratory distance of 786-O and ACHN cells (Figure 2G, 2H), but PLCL1 knockdown induced migratory capacity in the 769P cell line (Figure 2I–2L). Similar results were observed in the transwell assay. Together, these results suggest that PLCL1 represses tumour proliferation and invasion in RCC cells.

### PLCL1 promotes cancer cell apoptosis in RCC cells

**Figure 3:**
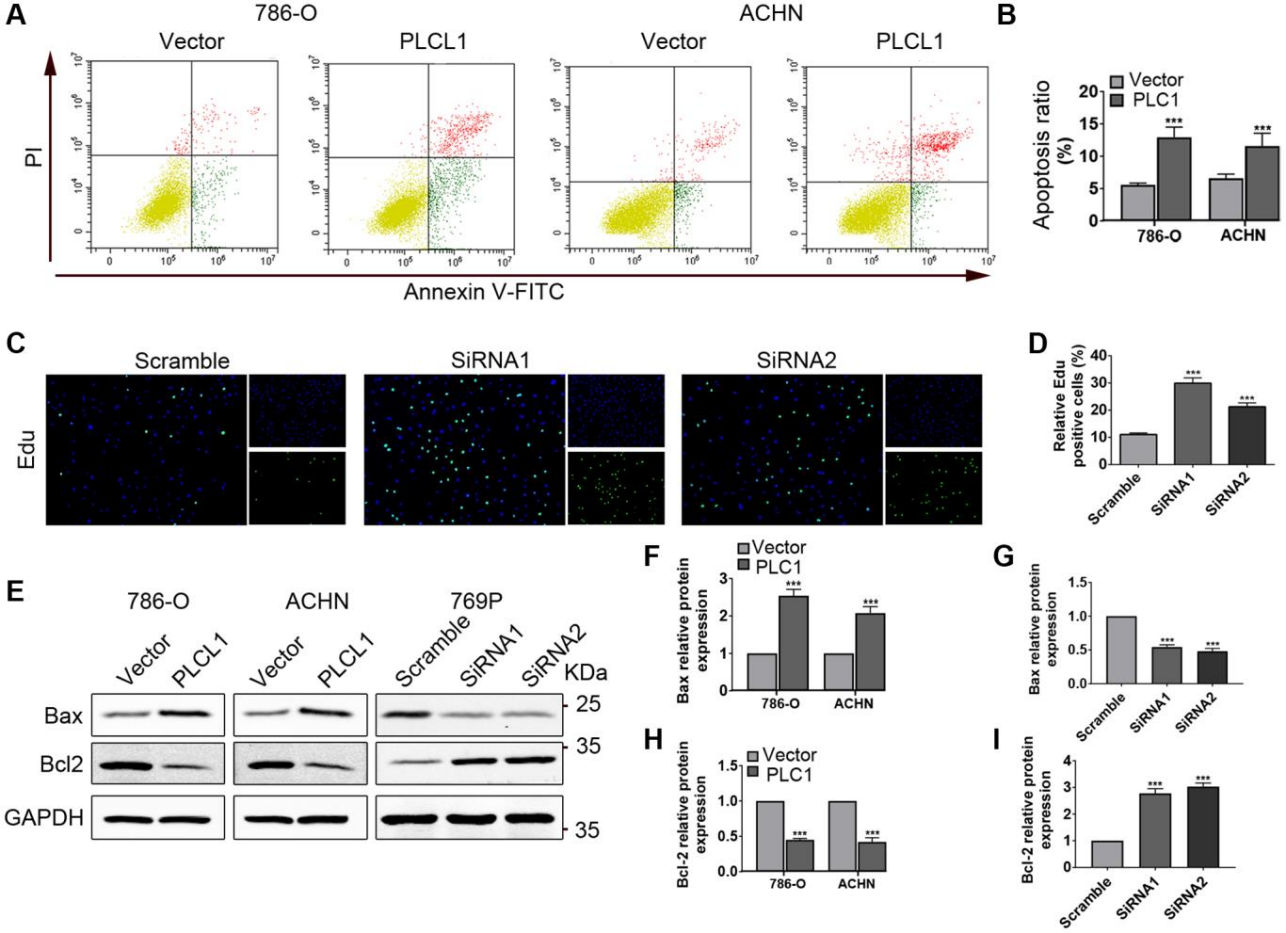
**PLCL1 induces apoptosis in RCC cells.** (**A**, **B**) Flow cytometry analysis was utilized to measure apoptosis in 786-O and ACHN cells transfected with PLCL1 or vector. (**C**, **D**) 769P cells were transfected with siRNA-PLCL1 #1, shRNA-PLCL1 #2 or siRNA-scrambled, and proliferation was examined using Edu kits, nuclei stained blue by DAPI, green for positive cells. Scale bar: 50 μm. (**E**–**I**) Representative western blots and quantification analysis of Bax and Bcl2 in RCC cells with different treatments. GAPDH was used as a loading control. Data are shown as the mean ± SE from three independent experiments. Student’s *t* test was performed to determine statistical significance between two groups. ^*^P < 0.05; ^**^P < 0.01; ^***^P < 0.001.

To further explore whether PLCL affects RCC apoptosis *in vitro*, flow cytometry and EdU analysis were performed. As indicated in Figure 3A, 3B, compared to the vector group, overexpression of PLCL1 significantly induced apoptosis of tumour cells, especially the early stage of apoptosis, while the ratio of proliferation was dramatically promoted in 769P cells treated with PLCL1 siRNA (Figure 3C, 3D). The Bcl-2 family has been documented to play a crucial role in the regulation of tumour cell apoptosis [20]. Therefore, two important regulators, Bax and Bcl2, were assessed using western blotting (Figure 3E and Supplementary Figure 3). As shown in Figure 3G, 3H, compared to that in the control cells, protein expression of Bax was increased, while levels of Bcl2 were decreased in 786-O and ACHN cells overexpressing PLCL1. Interestingly, the anti-apoptotic regulator Bcl2 exhibited the opposite results in the siRNA groups (Figure 3F–3I). Collectively, these data suggest that PLCL1 induces renal tumour cell apoptosis by upregulating Bax/Bcl2 expression.

### PLCL1 is involved in autophagy in RCC cells

**Figure 4:**
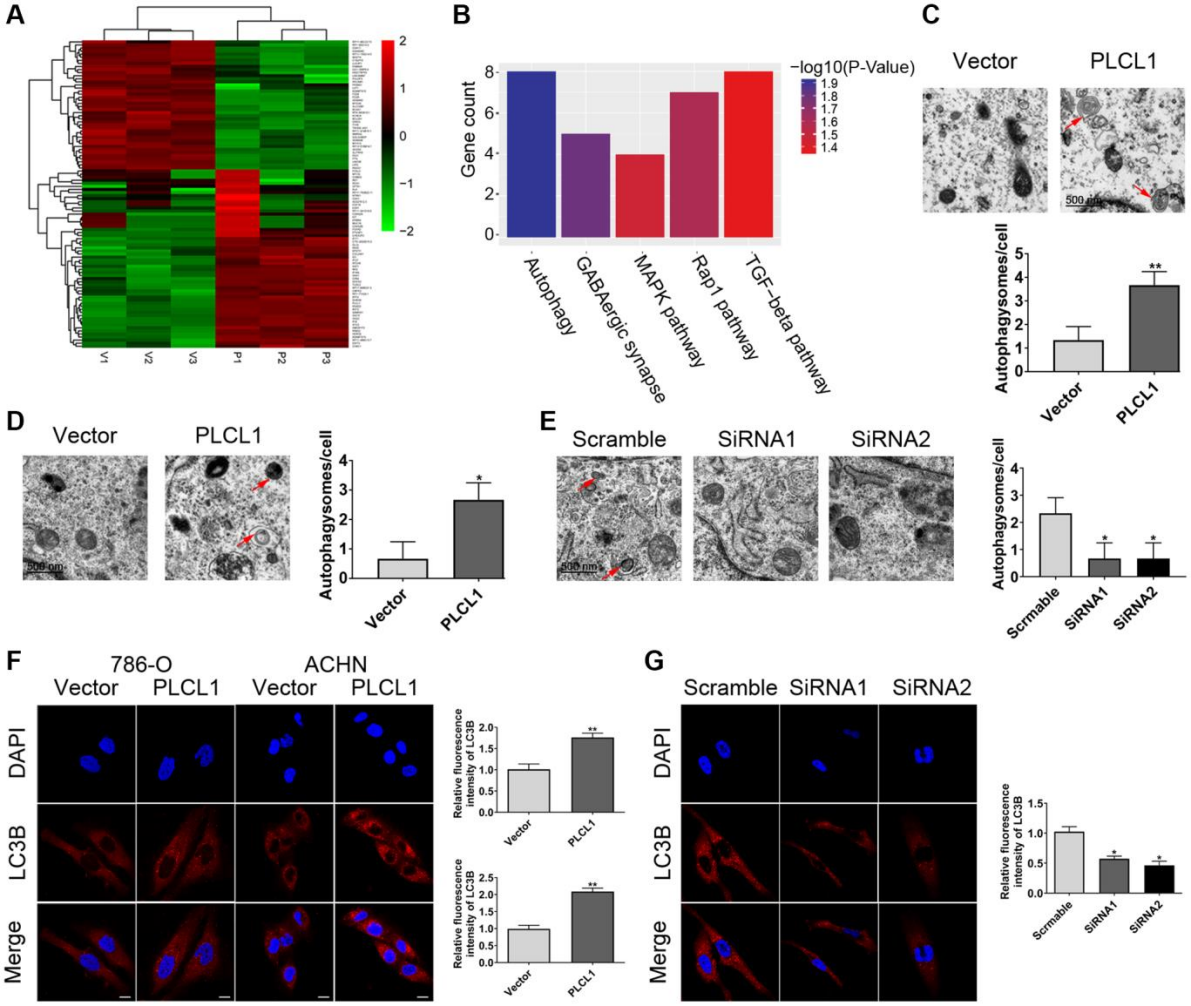
**PLCL1 facilitates autophagy in RCC cells.** (**A**) The heatmap of clustering analysis according to the sequencing of differentially expressed genes. (**B**) KEGG pathway enrichment analysis of the top five results. (**C**–**E**) Transmission electron microscopy indicating the formation of autophagosomes or autolysosomes in RCC cells. Red arrows: autophagosomes or autolysosomes. Scale bar: 1 μm. (**F**) 786-O and ACHN cells transfected with PLCL1 lentivirus and vector were subjected to immunofluorescence and quantitative analysis for LC3B. Scale bar, 20 μm. (**G**) Representative confocal images and quantitative analysis of LC3 dots in 769P cells transfected with siRNA-scramble, siRNA-PLCL1 #1, and shRNA-PLCL1 #2. Scale bar, 20 μm. Data are shown as the mean ± SE from three independent experiments. Student’s *t* test was performed to determine statistical significance between two groups. ^*^P < 0.05.

An increasing number of studies have demonstrated the crucial role of autophagy in RCC pathogenesis [21]. To determine whether PLCL1 affects autophagy during the development of RCC, 786-O cells transfected with PLCL1 or vector and were subjected to high-throughput RNA-sequencing (Figure 4A). KEGG pathway enrichment analysis of DEGs indicated that the top five pathways were autophagy, GABAergic synapse, MAPK signalling pathway, Rap1 signalling pathway and TGF-beta signalling pathway (Figure 4B). Interestingly, the transmission electron microscopy (TEM) results showed that PLCL1 significantly increases the numbers of autophagosomes/autolysosomes in 786-O and ACHN cells compared with those in the vector group (Figure 4C, 4D), but 769P cells with PLCL1 knockdown exhibited reduced accumulation of autophagosomes/autolysosomes compared with those in the scrambled group (Figure 4E). This autophagic phenotype was further supported by the immunofluorescence results of LC3B, an important marker of autophagy, which exhibited significant accumulation of LC3 puncta in the PLCL1 overexpression group (Figure 4F). Compared to the scrambled cells, knockdown of PLCL1 reduced autophagic vesicles in 769P cells (Figure 4G). These results all indicated that PLCL1 has an important relationship with autophagy.

### PLCL1 induces autophagic flux through activation of the AMPK/mTOR pathway *in vitro*

**Figure 5:**
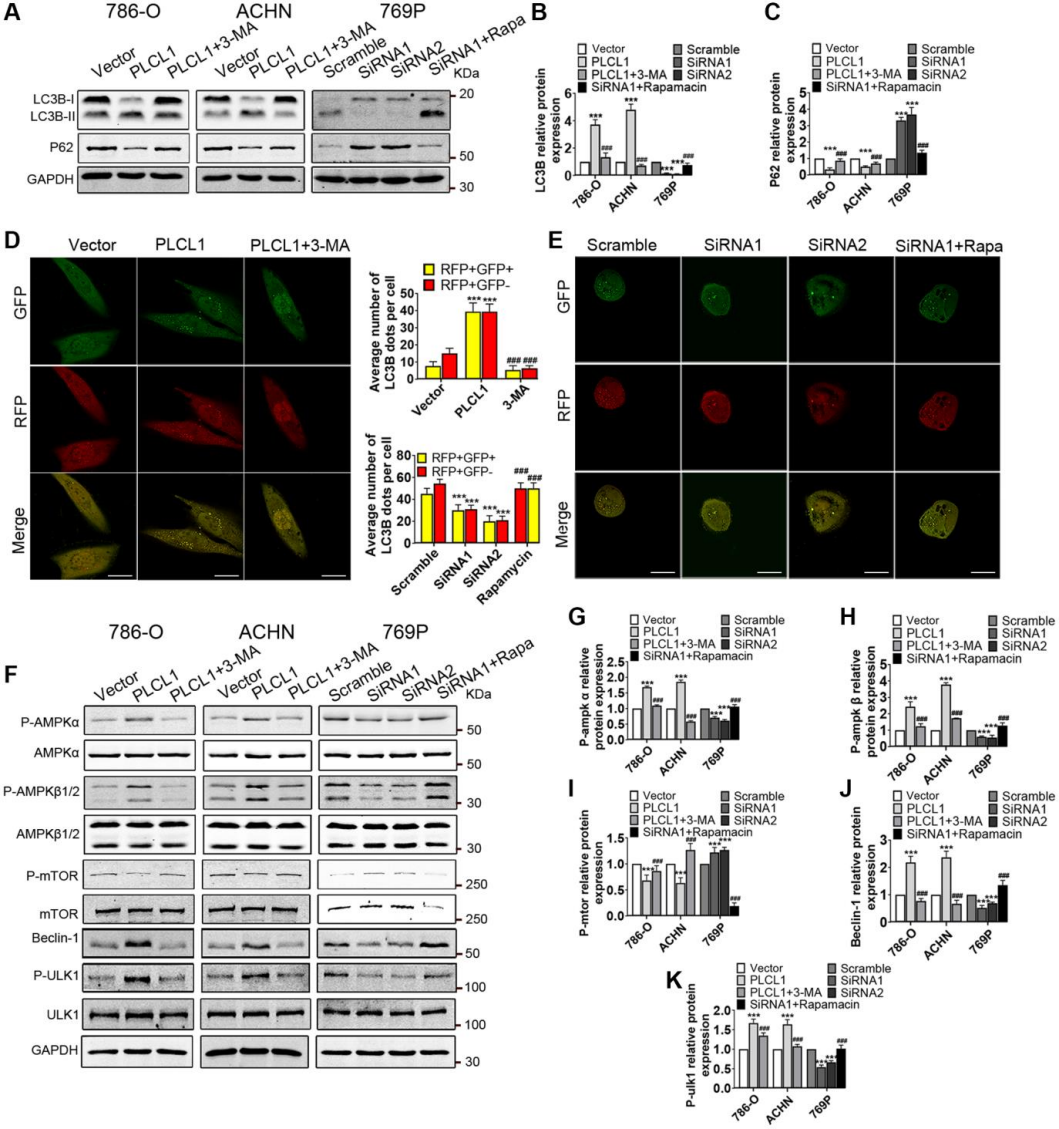
**PLCL1 promotes autophagic flux by regulating the AMPK/mTOR signalling pathway in RCC cells.** (**A**–**C**) Expression of LC3B and p62 in RCC cells with different treatments was examined using western blotting with quantitative analysis. GAPDH was used as a loading control. (**D**, **E**) 786-O and 769P cells transfected with GFP-mRFP-LC3B adenovirus were analysed using immunofluorescence. Autolysosome (red dots) and autophagosome (yellow dots) formation are shown using confocal microscopy and were quantitively analysed. Scale bar, 20 μm. (**F**–**K**) Representative western blotting and quantitative analysis of AMPK/mTOR signalling pathway-related proteins. Data are shown as the mean ± SE from three independent experiments. Student’s *t* test was performed to determine statistical significance between two groups. ^*^P < 0.05; ^**^P < 0.01; ^***^P < 0.001 versus vector or scramble group. ^#^*P* < 0.05; ^#^*P* < 0.01; ^#^*P* < 0.001 versus cells overexpressing PLCLl or siRNA1.

To investigate whether PLCL1 promotes autophagic flux, RCC cells were treated with 3-MA or rapamycin, and western blotting was performed to determine the protein levels of microtubule-associated protein 1 light chain 3β (LC3B) and sequestosome 1 (SQSTM1 or p62). As shown in Figure 5A, PLCL1 markedly increased levels of LC3B-II and decreased expression of p62; however, protein expression levels were decreased by treatment with 3-MA, an autophagy inducer. Interestingly, silencing PLCL1 using siRNA1 and siRNA2 resulted in the opposite results for LC3B-II and p62 compared to the scrambled group (Figure 5B, 5C). These results suggest that autophagy is ibzinitiated. Autophagic flux was then assessed in 786-O, ACHN and 769P cells transfected with autophagic adenovirus using a fluorescence confocal microscope. As shown in Figure 5D and Supplementary Figure 4, in PLCL1-overexpressing cells, the intensity of red puncta (autolysosomes) and yellow puncta (autophagosomes) per cell was remarkably induced and primarily accumulated in the cytoplasm compared to those in the vectors, while this change was hampered by treatment with 3-MA, suggesting that autophagic flux is promoted by PLCL1. Of note, in PLCL1 knockdown with siRNA and siRNA2 cells, the intensity of red and yellow dots was impaired compared to scrambled 769P cells, while rapamycin reversed this alteration (Figure 5E). Cumulative evidence indicates that the AMPK/mTOR signalling pathway plays a crucial role in the process of autophagy. To determine whether the AMPK/mTOR pathway has an effect on PLCL1-induced autophagy in RCC, AMPK/mTOR pathway-related proteins were examined using western blot analysis (Figure 5F). As shown in Figure 5G–5K, compared to the vector group, overexpression of PLCL1 significantly enhanced the levels of P-AMPKα, P-AMPKβ1/2, Beclin-2 and P-ULK1 and decreased the levels of P-mTOR in 786-O and ACHN cells, whereas this alteration was restored after treatment with 3-MA. These results further supported by the observation that the AMPK/mTOR pathway was activated by PLCL1 but inhibited by rapamycin in 769P cells.

### PLCL1 regulates autophagy by interacting with DEPP in RCC cells

**Figure 6:**
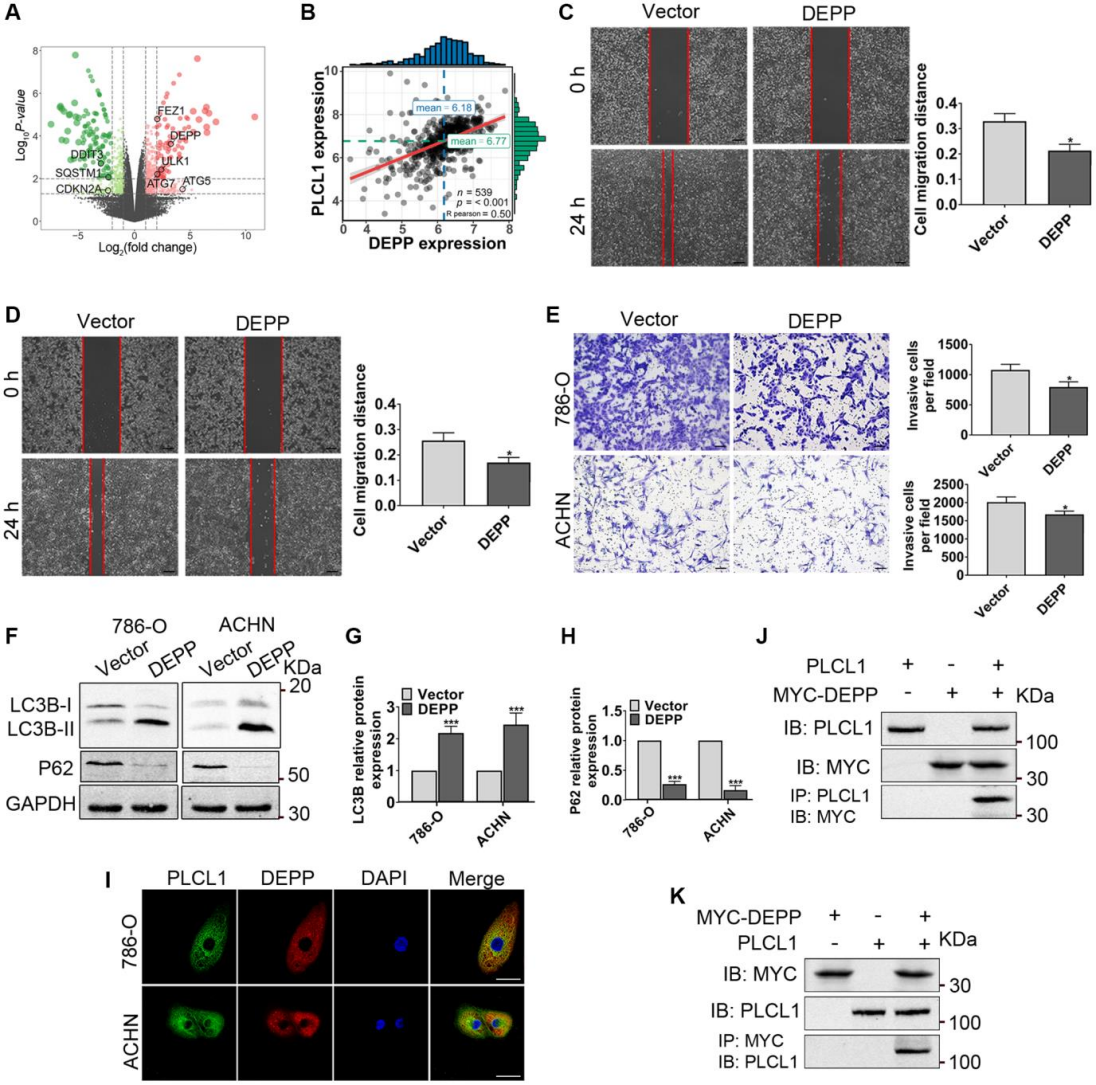
**PLCL1 promotes autophagy by interacting with DEPP.** (**A**) Volcano plot of autophagy-related DEGs (eight candidates, black colour) in 786-O cells with PLCL1 overexpression and vector cell sequencing. The red dots in the volcano represent upregulated DEGs, and the green dots represent downregulated DEGs, while black dots represent non-significant DEGs. (**B**) The association between PLCL1 and DEPP was calculated using Pearson analysis. (**C**, **D**) Wound healing assays were used to determine the role of DEPP in RCC *in vitro* and quantification analyses of the results. Scale bar: 100 μm. (**E**) Transwell assays were performed in 786-O and ACHN cells transfected with DEPP or vector control. Scale bar: 100 μm. (**F**–**H**) Western blots of LC3B and P62 were performed in 786-O and ACHN cells transfected with DEPP or vector control. (**I**) 786-O and ACHN cells were immunofluorescently stained with PLCL1 (green) and DEPP (red) antibodies to assess colocalization using a confocal microscope. Scale bar, 20 μm. (**J**, **K**) Interaction between PLCL and DEPP in 786-O cells. The coimmunoprecipitates were utilized for western blotting with anti-PLCL1 and anti-MYC antibodies. Data are shown as the mean ± SE from three independent experiments. Student’s *t* test was performed to determine statistical significance between two groups. ^*^P < 0.05; ^**^P < 0.01; ^***^P < 0.001 versus vector group.

The above results reveal an important relationship between PLCL1 and the AMPK/mTOR pathway in regulating autophagy *in vitro*. Therefore, autophagy-related DEGs from the mRNA sequencing results were selected to further elucidate the potential mechanism between PLCL1 and the AMPK/mTOR pathway in RCC cells. As shown in Figure 6A, eight were selected, five upregulated and five regulated genes, and one of the DEGs, DEPP, was significantly positively correlated with PLCL1, exhibiting the highest correlation coefficient (R^2^ = 0.51, *P* < 0.001) in mRNA expression based on Pearson’s correlation of TCGA RCC tumours (Figure 6B). To explore the effect of DEPP on RCC progression, 786-O and ACHN cells were transfected with DEPP and vector control lentiviruses, and transfection efficiency was confirmed by RT–qPCR and western blotting (Supplementary Figure 5). The results of wound healing assays showed that DEPP remarkably inhibited migration capacity compared that in vector cells (Figure 6C, 6D). Likewise, the transwell assay indicated that compared to the control group, numbers of 786-O and ACHN cells were significantly reduced in the DEPP overexpression group (Figure 6E). Moreover, the western blotting results revealed that DEPP significantly increased the conversion of LC3B-I to LC3B-II but decreased the expression of P62, implying that DEPP might be correlated with autophagy in RCC (Figure 6F–6H). To understand the relationship between PLCL1 and DEPP, immunofluorescence and coimmunoprecipitation assays were performed. As shown in Figure 6I, PLCL1 was expressed in the cytoplasm, DEPP was primarily expressed in the cytoplasm, and the colocalization of PLCL1 and DEPP occurred mostly in the cytoplasm, illustrating that PLCL1 is correlated with DEPP in RCC cells. Then, 786-O cells were subjected to immunoprecipitation for PLCL1 and DEPP. As shown in Figure 6J, 6K and Supplementary Figure 6, reciprocal immunoprecipitation further supported the notion that PLCL1 interacts with DEPP. Meanwhile, we also found that PLCL1 co-localized with LC3BII (Supplementary Figure 7). Collectively, these results demonstrate that PLCL1 promotes autophagy by interacting with DEPP.

### PLCL1 inhibits RCC growth *in vivo*

**Figure 7:**
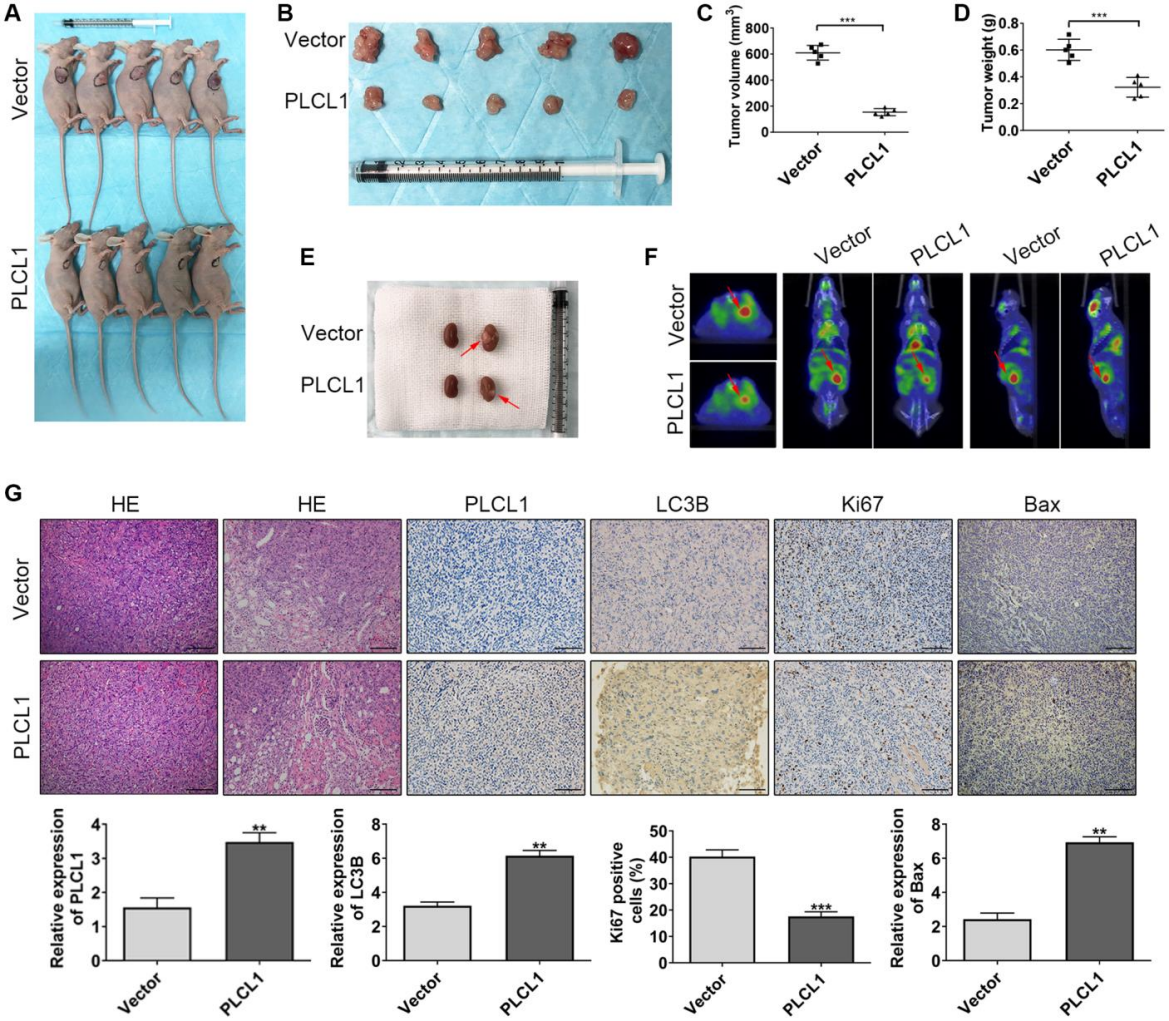
**PLCL1 suppresses RCC tumour proliferation *in vivo*.** (**A**, **B**) Representative images of PLCL1 and PLCL1-control RCC tumour growth in Balb/c nude mice. The tumour volume (**C**) and weight (**D**) in the xenograft tumour model are shown. (**E**) Representative images of renal orthotopic models between PLCL1 and vector control samples. (**F**) Representative PET scan results of the mouse orthotopic model. (**G**) HE, immunohistochemistry and quantitative analyses of PLCL1, LC3B, Ki67 and Bax in mouse tumour specimens. Each group *n* = 5. Student’s *t* test was performed to determine statistical significance between two groups. Scale bar: 100 μm. ^**^P < 0.01, ^***^P < 0.001.

To further assess the antitumour function of PLCL1, Balb/c nude mice were used. As shown in Figure 7A–7D, compared to the vector group, PLCL1 significantly decreased the tumour volume and weight derived from the xenograft tumour model. Moreover, 786-O cells transfected with lentivirus PLCL1 or vector were injected into the left kidney of mice to establish an orthotopic tumour model (Figure 7E, red arrow). Interestingly, consistent with findings in the orthotopic model, positron emission computed tomography exhibited similar results in the horizontal, coronal and sagittal planes (Figure 7F, red arrow). Moreover, compared to vector tissues, HE of the PLCL1 group samples exhibited fewer areas of nuclear pleomorphism, and the results of IHC staining revealed increased LC3B and Bax expression levels and decreased levels of Ki67 compared to those in vector group tissues from the xenograft tumour model (Figure 7G). These data suggest that PLCL1 suppresses RCC tumour proliferation, further confirming the results *in vitro*.

## DISCUSSION

Autophagy is an incessant and dynamic process that is involved in the clearance of damaged and necrotic components of cells, sustaining metabolic homeostasis [9]. Many studies have characterized autophagy as a crucial regulatory mechanism in the progression of RCC. One study showed that LC3B-dependent autophagy was activated in proliferating RCC cell lines, whereas silencing LC3B levels decreased tumour volume in a RCC xenograft tumour model [22]. Deng et al. reported that low levels of LC3B-II were significantly correlated with poor survival in RCC patients [23]. Consistent with those studies, our findings showed that overexpression of PLCL1 induces levels of LC3B-II and initiates autophagic flux, impairing the capacity of proliferation and invasion in RCC cell lines. Our data suggest that LC3B mediated by PLCL1 is an important regulator in the pathogenesis of RCC. In addition, the AMPK/mTOR signalling pathway has been extensively investigated and reported in a series of diseases because it plays a crucial role in the regulation of autophagy, especially in tumour progression [16, 24]. A previous study indicated that activation of AMPK represses mTOR activity and induces phosphorylation of the targeted autophagy-related gene ULK1, initiating autophagy [21]. Previous studies have indicated that activation of the AMPK/mTOR pathway inhibits the epithelial-mesenchymal transition, repressing tumour cell growth and metastasis. Suppression of the AMPK/mTOR pathway decreases autophagic flux and reduces the number of autophagosomes, resulting in the evolution of docetaxel-acquired resistance in prostate tumour cells [25]. Our data showed that in RCC cells, activation of the AMPK/mTOR signalling pathway by enhancing PLCL1 levels remarkably repressed cell proliferation and invasion and promoted apoptosis, whereas suppression of the AMPK/mTOR pathway by silencing PLCL1 contributed to reduced production of autophagosomes and enhanced cell growth. These data illustrate that AMPK/mTOR pathway-mediated autophagy plays a crucial role in the development of RCC.

PLCL1 is a significant member of the phospholipase C family, participating in a series of physiological-pathological responses that affect the occurrence and progression of disease [26]. Previous findings have primarily focused on the structure of PLCL1, indicating that genetic single nucleotide polymorphisms of PLCL1 are correlated with hip bone size in females and idiopathic inflammatory myopathies [6, 7]. A recent study revealed that PLCL1 has a critical effect on maintaining a balance between metabolism and RCC by mediating lipid metabolic gene ubiquitination levels and consuming lipids without producing adenosine triphosphate energy, inhibiting tumour growth [8]. Consistent with those discoveries, our data from CCK8, wound healing and transwell assays demonstrated that PLCL1 represses the proliferation, migration and invasion capacities of RCC cells. Simultaneously, the results of orthotopic xenograft models also showed that PLCL1 decreased tumour volume and weight compared to those in control mice. Unlike previous findings, in the present study, by overexpressing PLCL1 *in vitro*, we demonstrated that high levels of PLCL1 affect autophagy activation, repressing the RCC progression. 786-O, ACHN and 769P cells were utilized to elucidate a new mechanism by which PLCL1 activates the AMPK/mTOR pathway, induces autophagosome formation, suppresses autophagic degradation and promotes proapoptotic protein expression, suppressing progression and accelerating apoptosis in RCC. In addition. Our bioinformatics findings were based on GEO and TCGA, two major databases of tumour pathogenesis, whereas previous findings were derived from metabolic databases. However, although the mechanism and research methods are slightly different, both findings support that PLCL1 may function as a tumour suppressor in the occurrence and progression of RCC.

DEPP was first identified as a protein related to progesterone in endometrial stromal cells [27, 28]. Accumulating evidence has shown that DEPP is positively linked with breast cancer and has a connection with energy deprivation and ionizing radiation [29–31]. Specifically, levels of DEPP are elevated in response to starvation and oxidative stress, which in turn promoted reactive oxygen species (ROS) accumulation, resulting in mitochondrial dysfunction and apoptosis [32]. Overexpression of DEPP by lentivirus from our study in 786-O and ACHN cells is in line with previous evidence, which indicated that DEPP markedly decreases the migration distance and invasive number of RCC cells in wound healing and transwell assays. Moreover, one of the important functions of DEPP, as discovered in previous studies, is to provoke autophagy [30, 33]. In hepatocellular carcinoma and neuroblastoma cell lines, overexpression of DEPP promoted autophagic flux, whereas knockdown of DEPP inhibited this alteration compared to control cells [30]. LC3B has been proven to be required for the formation of autophagosomes at the beginning of autophagic flux [34]. Similar findings in our study showed that DEPP remarkably enhanced the conversion of LC3B-I to LC3B-II accompanied by a decline in the expression of P62, implying that DEPP also promotes autophagy in RCC. Moreover, our findings revealed that the involvement of PLCL1 in the regulation of autophagy not only through AMPK/mTOR signalling, but through its interaction with DEPP.

Our data reveal that PLCL1 functions as a suppressor in RCC progression and that there is a positive correlation between PLCL1 and patient prognosis. The mechanisms of the suppressive role may occur through activating the AMPK/mTOR pathway along with interacting with DEPP, initiating autophagy and thereby inducing apoptosis. However, it is unclear whether and how DEPP influences the AMPK/mTOR pathway in the progression of RCC, which we will investigate in the future. Our present study demonstrates that PLCL1 may be a promising biomarker for the diagnosis of RCC and that targeting PLCL1 would be an effective treatment for RCC.

## Supplementary Materials

Supplementary Figures

Supplementary Table 1
