# Supplementary material for: PLCL1 suppresses tumour progression by regulating AMPK/mTOR-mediated autophagy in renal cell carcinoma
Source: Aging (Albany NY). 2023 Oct 6;15(19):10407–27. doi: 10.18632/aging.205085 (PMC10599749; doi:10.18632/aging.205085)
Supplement: Supplementary Figures [file aging-15-205085-s001.pdf]

SUPPLEMENTARY FIGURES

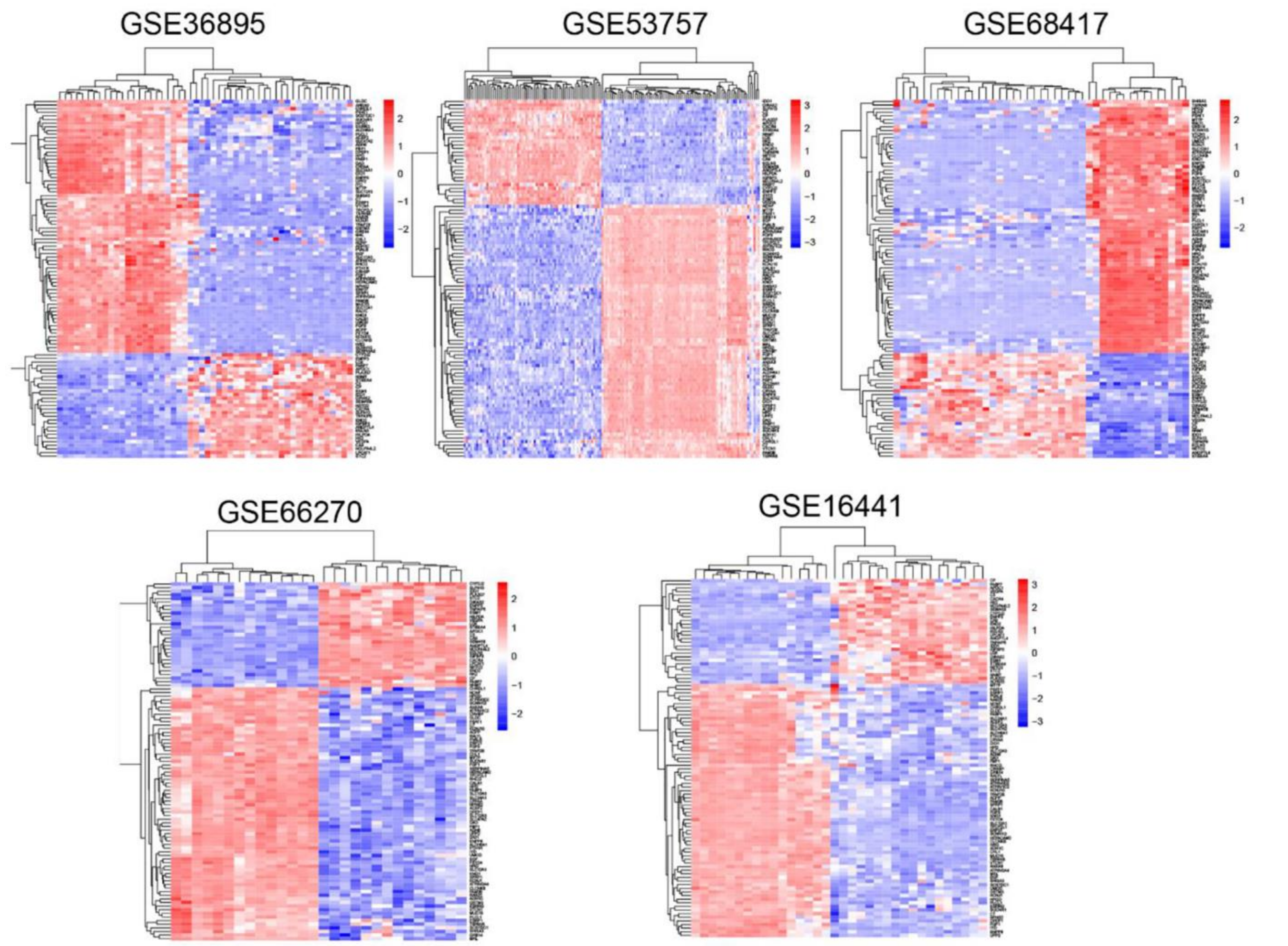

Supplementary Figure 1. Heatmap of 99 genes in five datasets. Clustering analysis of the 99 genes in each independent dataset. Each column represents a sample and each row represents the expression level of a gene. The color scale represents the raw Z score ranging from blue (low expression) to red (high expression). Dendrograms by each heatmap correspond to the hierarchical clustering by expression of the 99 mRNA.

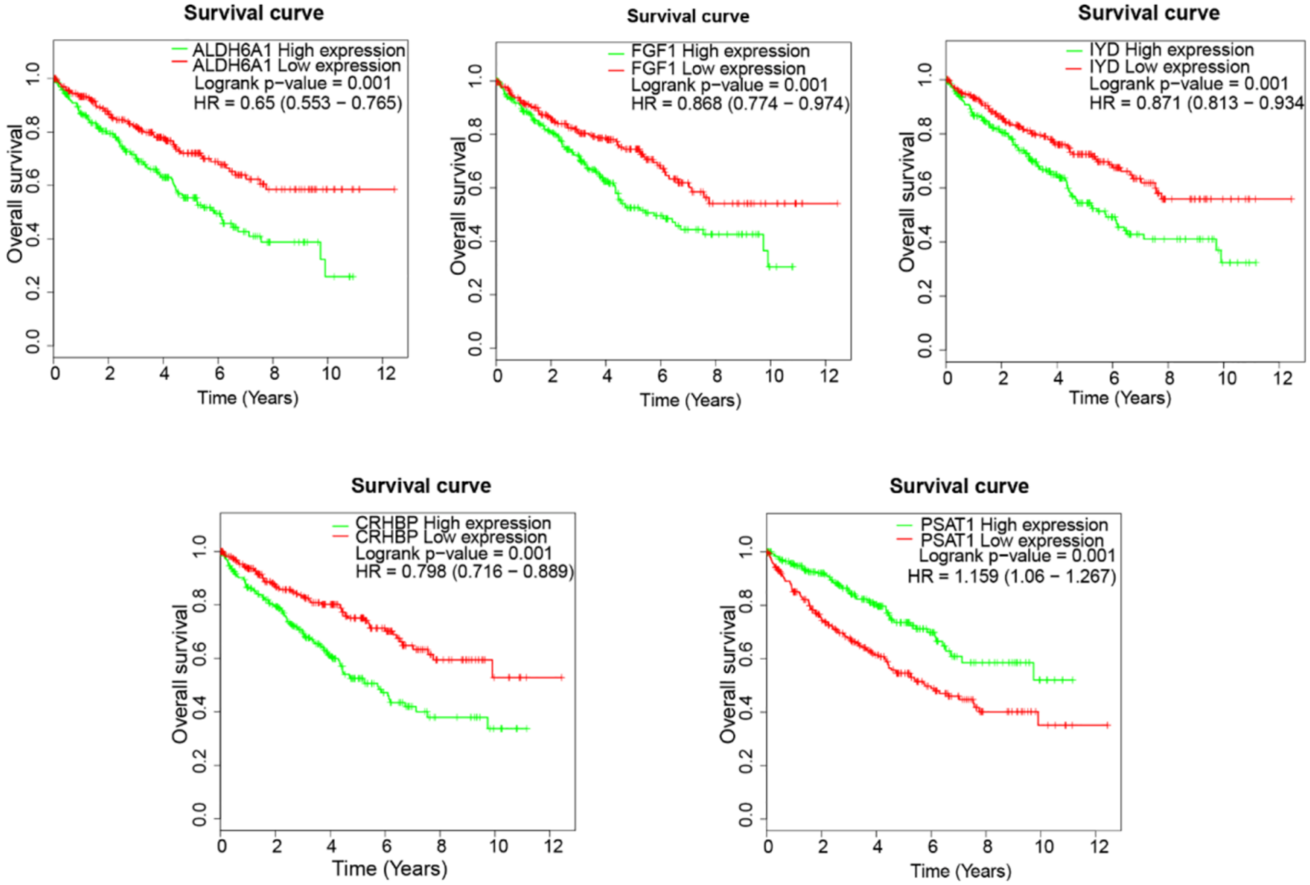

Supplementary Figure 2. The Kaplan-Meier curve of ccRCC patients in TCGA grouped based on the median levels of each gene. Abbreviation: HR: hazard ratio.

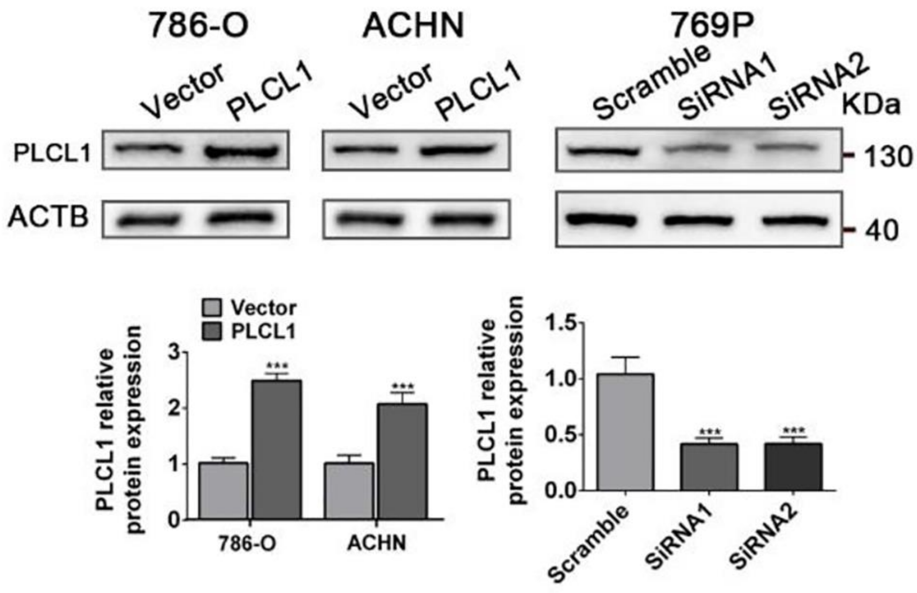

Supplementary Figure 3. Representative western blots and quantification analysis of PLCL1 in ccRCC cells transfected with Vector or PLCL1-targeted lentivirus. ACTB was used as a loading control. $^{*}P < 0.05$; $^{**}P < 0.01$; $^{***}P < 0.001$, versus the Vector group.

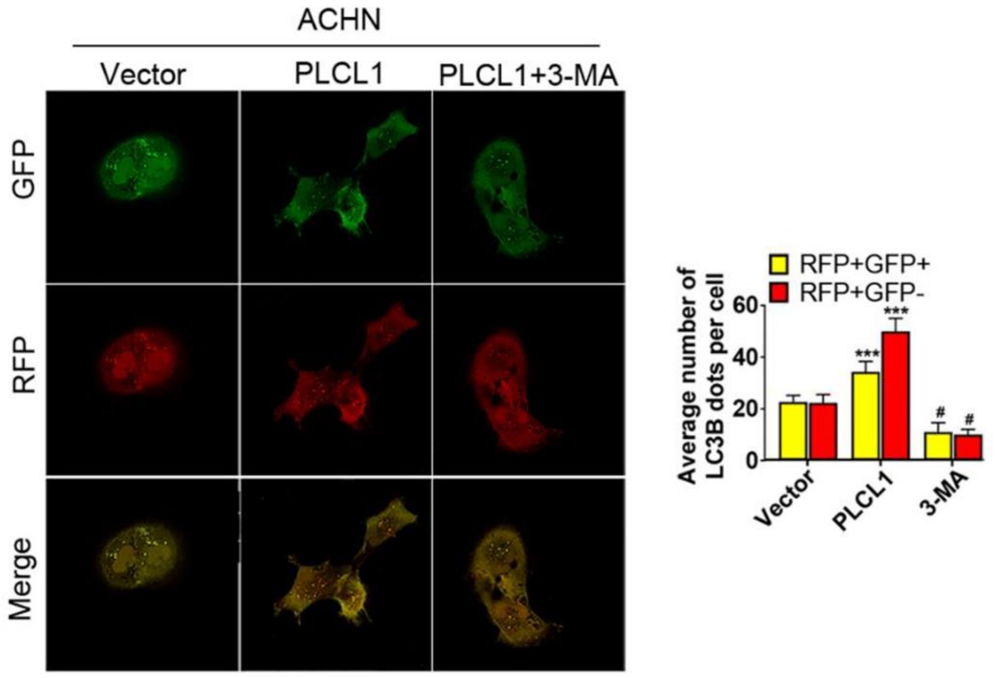

Supplementary Figure 4. ACHN cells transfected with GFP-mRFP-LC3B adenovirus were analysed using immunofluorescence. Autolysosome (red dots) and autophagosome (yellow dots) formation are shown using confocal microscopy and were quantitively analysed. Scale bar, 20 μm.

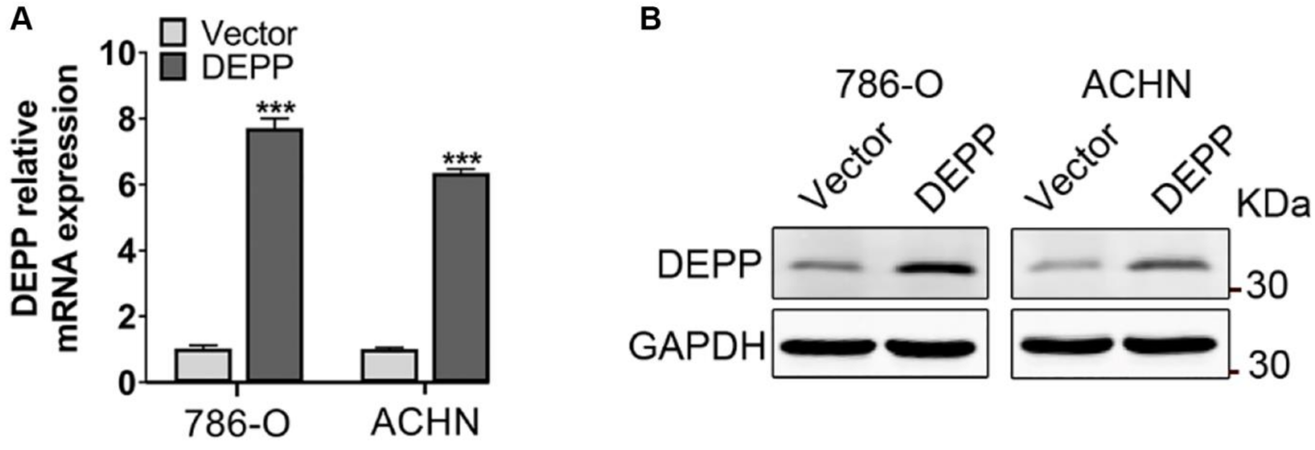

Supplementary Figure 5. Transfection efficiency of ccRCC cells. 786-O and ACHN were transfected with DEPP lentivirus and vector and analyzed by RT-qPCR and western blotting (**A**, **B**).

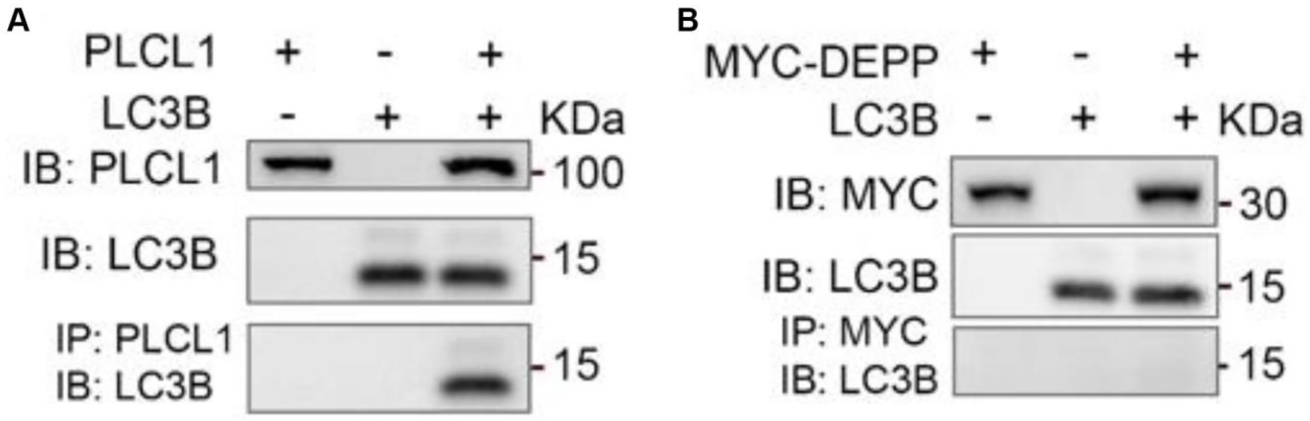

Supplementary Figure 6. (**A**, **B**) Interaction between PLCL and LC3B or DEPP and LC3B in 786-O cells. The coimmunoprecipitates were utilized for western blotting with anti-PLCL1, anti-LC3B and anti-MYC antibodies.

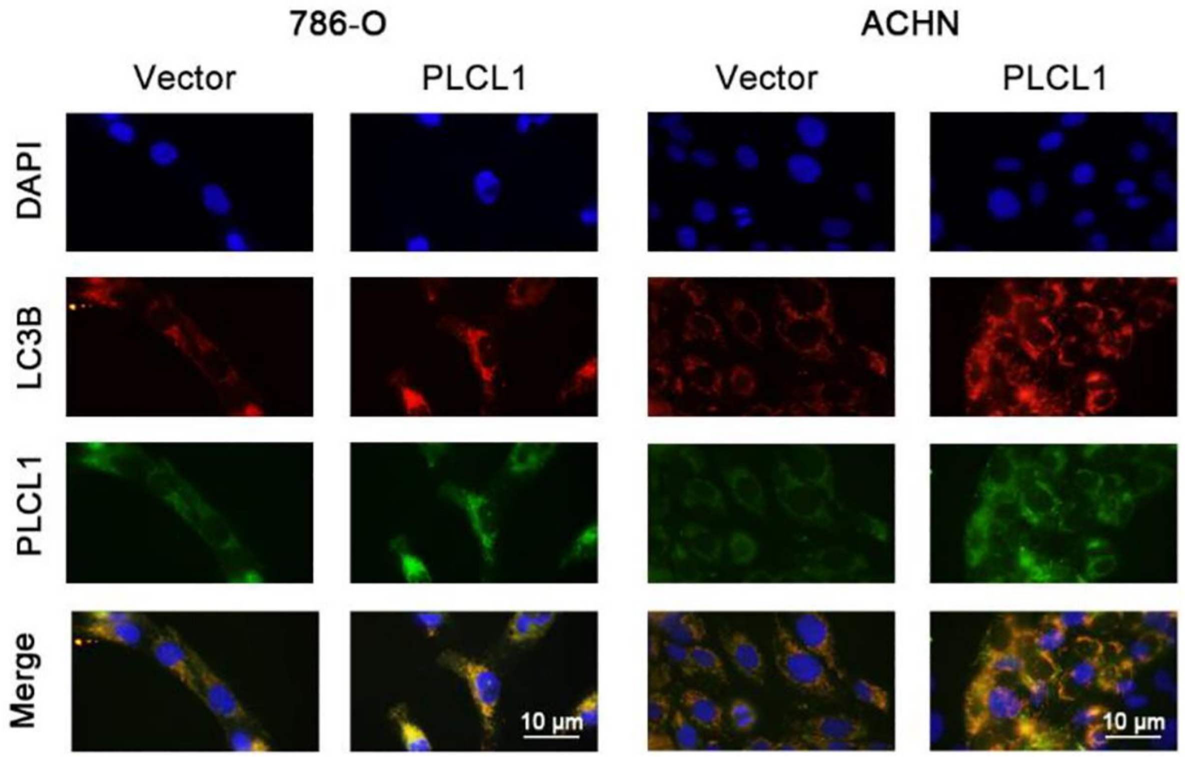

Supplementary Figure 7. Co-localization and expression of LC3B (red) and PLCL1 (green) in 786-O and ACHN Vector and PLCL1 overexpressing cells were examined by fluorescence microscopy.
