# Supplementary material for: PLCL1 suppresses tumour progression by regulating AMPK/mTOR-mediated autophagy in renal cell carcinoma
Source: Aging (Albany NY). 2023 Oct 6;15(19):10407–27. doi: 10.18632/aging.205085 (PMC10599749; doi:10.18632/aging.205085)
Supplement: Supplementary Table 1 [file aging-15-205085-s002.pdf]

SUPPLEMENTARY TABLE

Supplementary Table 1. Antibodies used in this study.

Antibody	WB	IHC	IF	Company and catalog
PLCL1	1:1000	1:100	1:50	Abcam, ab157200
Bax	1:1000	1:100	1:50	Cell Signaling, 5023S
Bcl2	1:1000	1:100		Abcam, ab182858
P-mTOR	1:1000			Abcam, ab109268
mTOR	1:1000			Cell Signaling, 2983S
P-AMPKα	1:1000			Cell Signaling, 2535S
AMPKα	1:1000		1:50	Cell Signaling, 5831S
P-AMPKβ1	1:1000			Cell Signaling, 4186S
P-AMPKβ2	1:1000			Cell Signaling, 82791S
AMPKβ1/2	1:1000			Cell Signaling, 4150S
Beclin-1	1:1000			Cell Signaling, 3495S
P-ULK1	1:1000			Cell Signaling, 37762S
ULK1	1:1000			Cell Signaling, 8054
LC3B	1:1000	1:100		Abcam, ab192890
P62	1:1000		1:50	Cell Signaling, 39749S
Ki67		1:100		Abcam, ab15580
DEPP		1:100	1:50	Proteintech, 25833-1-AP
GAPDH	1:5000			Abcam, ab8245
IgG	1:1000			Abcam, ab172730
MYC-tag	1:1000			Abcam, ab9106
β-actin	1:5000			Abcam, ab8226
